# Assessing SRTM one Arc second DEM accuracy for small dam volume-elevation curves using terrain metrics

**DOI:** 10.1038/s41598-025-30483-7

**Published:** 2025-12-27

**Authors:** Peshawa Bakhtyar Salih Ahmed, Nawbahar Faraj Mustafa, Shvan Fars Aziz, Nadhir Al-Ansari

**Affiliations:** 1 College of Engineering- Water Resources Department, University of Sulaimani, Sulaimaniyah, Iraq; 2 Department of Civil, Environmental and Natural Resources Engineering , Lulea University of Technology, Lulea, Sweden

**Keywords:** One-Arc-Second SRTM DEM, Small dam, Terrain ruggedness index (TRI), Sensitivity analysis, Volume-Elevation curve, Climate sciences, Environmental sciences, Hydrology, Natural hazards

## Abstract

Accurate reservoir storage estimation is fundamental to sustainable water resources management; however, small dam projects are often hindered by the prohibitive costs and time required for high-precision topographic surveys. In this study, a rigorous validation of freely available one-arc-second SRTM DEMs was conducted as an alternative approach for estimating volume-elevation relationships at ten small dams in Iraq. High-precision surveys served as benchmarks, enabling statistical validation of DEM-derived estimates using absolute relative error (ARE), root mean square error (RMSE), mean absolute error, and the coefficient of determination (R²). Reservoir basin morphology was further characterised through planimetric indices, including area-to-volume ratio (AVR), shape factor, and solidity. In parallel, terrain complexity within a 5 km buffer zone was quantified using slope variability, curvature, vector ruggedness measure (VRM), and terrain ruggedness index (TRI). A strong structural agreement was demonstrated (R² > 0.98), although substantial variation in volumetric precision was observed. A global sensitivity analysis using the Morris Method identified the standard deviation of the Terrain Ruggedness Index (TRI) as the dominant predictor of accuracy, with µ* values of 83–86, while all other metrics showed minimal influence (µ* ≈ 0–24). These results establish a clear accuracy threshold for one-arc-second SRTM DEMs: they are sufficiently reliable for preliminary planning (< 20% error) in low-ruggedness terrain (TRI SD < 0.1) but become highly unreliable in rugged landscapes, where errors exceed 150% (TRI SD > 0.1). These findings provide a predictive framework for assessing DEM suitability, supporting the integration of satellite topography into small-scale reservoir planning.

**Supplementary Information:**

The online version contains supplementary material available at 10.1038/s41598-025-30483-7.

## Introduction

Topography is a key land-surface feature that significantly impacts the water balance within a watershed and the capacity of reservoirs^1^. Surveying the topography of reservoir and dam locations is essential for determining site suitability for dam construction; however, it is a time-consuming and expensive process^2^. Highly accurate Digital Elevation Models DEMs are not commonly available, particularly in developing countries^3^. Meanwhile, one-arc-second-resolution DEMs are freely available and easily downloadable, offering more efficient and cost-effective alternatives^3^. Numerous studies have used DEMs and Remote Sensing technologies to estimate water volumes in large reservoirs across various global locations^4–13^. Moreover, extensive research has been conducted to ascertain the volume-elevation data for the reservoirs. For instance, Ahmet Irvem generated a DEM of the Buyuk Karacay basin from a topographic map using the Ripple method^14^. In a subsequent 2020 study, Irvem investigated the effects of DEM resolution on the determination of dam storage capacity using Geographic Information Systems (GIS). He concluded that a 10-meter resolution provides the most accurate estimates of reservoir volume^15^.

Yao Li et al. presented a framework for deriving reservoir volume-area-elevation data from TanDEM-X data, integrating volume values from the top to bottom layers to generate the final data^16^. Mengfei Mu et al. combined the Global Reservoir and Dam (GRanD) database with Landsat-based global surface water extent (GSW) data to derive area-volume-elevation data of reservoirs, and the resulting data were validated with in situ data from the US and China, showing accurate results for reservoirs larger than one km² and with various shapes^17^.

A common thread in these studies is their focus on medium to large-sized reservoirs, where the larger spatial scale diminishes the relative impact of DEM vertical error on volumetric calculations^18^.

While research validating the use of one-arc-second Digital Elevation Models (DEMs) for calculating volume-elevation data in small reservoirs is limited, this study seeks to address this gap by evaluating and validating the effectiveness of one-arc-second DEMs for determining reservoir volume-elevation data. The primary objective is to establish one-arc-second DEMs as a feasible and efficient alternative to traditional on-ground surveying techniques during the feasibility phase of dam locations.

Therefore, this study moves beyond generic vertical accuracy assessment to address a more targeted research problem: Quantifying the suitability of one-arc-second SRTM DEMs for generating volume-elevation curves for small dams and developing a predictive framework to pre-assess their expected accuracy based on terrain characteristics. The SRTM DEM was selected for this study because it remains the most extensively used and cited global DEM in the scientific literature. Leveraging this widely recognised DEM ensures that the study’s methodology can be seamlessly replicated and that the results are comparable to existing and future research.

The primary objectives of this work are:

1. To perform a rigorous, site-specific validation of SRTM-derived reservoir volumes against high-precision GPS survey data across ten small dams.
2. To investigate the relationship between volumetric estimation error and a comprehensive suite of terrain complexity metrics, both within the reservoir basin and its surrounding territory.
3. To identify, through global sensitivity analysis, the dominant terrain parameter controlling volumetric accuracy.
4. To establish clear, quantifiable thresholds that guide practitioners on the expected reliability of SRTM DEMs for small dam planning based on local terrain ruggedness.

The novelty of this research lies in its integrated methodology that couples volumetric error analysis with advanced terrain morphometry and sensitivity analysis, specifically tailored to the small-reservoir domain. Unlike previous work, this study not only reports error statistics but also provides a practical and predictive framework. By demonstrating that the standard deviation of the Terrain Ruggedness Index (TRI) in a 5 km buffer zone explains over 80% of the variance in error, we provide a scientifically grounded tool for pre-project assessment. This contribution is pivotal for advancing the integration of satellite-based topography into the feasibility phase of small-scale water resource projects, particularly in data-scarce regions, while clearly defining the critical limits of its applicability.

### Study area

**Fig. 1:**
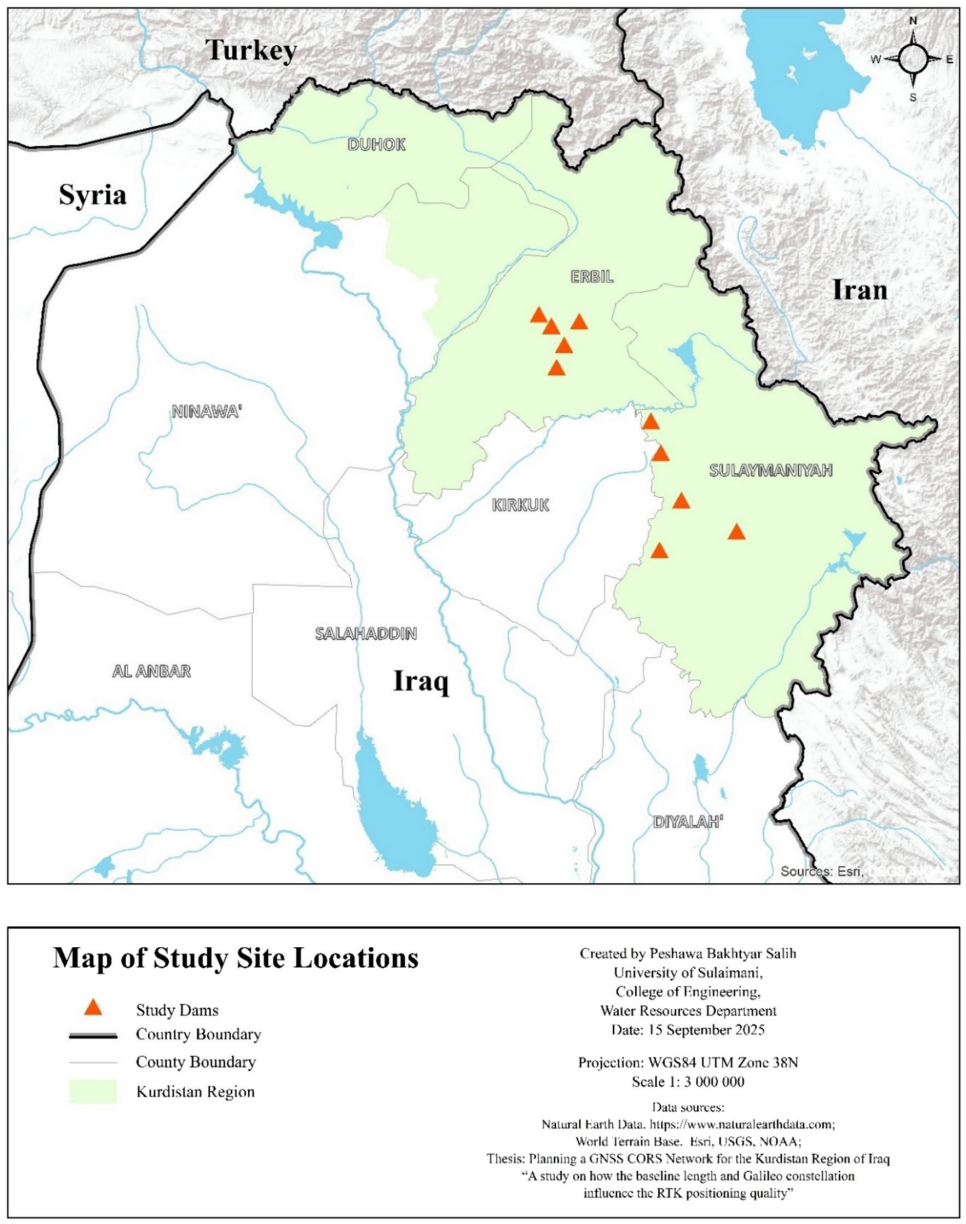
Map of the Study Area.

Data from on-ground surveying with GPS were gathered from the regional water resources authorities for ten locations in Erbil and Sulaymaniyah governorates, the Kurdistan Region of Iraq, as shown in Fig. 1. The study area encompasses these two distinct governorates—Erbil, situated on a vast alluvial plain with gentle topography, and Sulaymaniyah, located within a rugged, mountainous geologic zone. This intentional selection provides a diverse range of terrain complexities, allowing the study to evaluate DEM performance across varying geomorphological conditions. Reservoir volume-elevation data were compiled using the prismatic method^19^. The coordinates of the small dams are provided in Table 1.

**Table 1:** The location of small dams in UTM coordinates, with heights and reservoir volumes.

No.	Location	Dam name	Easting	Northing	Dam height m	Reservoir volume m^3^
1	Erbil	Kollak	424293.3	4,012,323	11	160,000
2		Bneslawa	430,726	4,002,579	20	2,200,000
3		Mzoryan	426,795	3,991,235	14	365,000
4		Klkan	417989.7	4,018,297	10	250,000
5		Tarin	438,509	4,014,585	15	131,546
6	Sulaymaniyah	Hamza Romi	518,813	3,906,954	14	190,000
7		Serchnar	474,758	3,963,599	18	160,000
8		Nwrey Serw	490,349	3,922,875	15	365,000
9		Zarda	479,040	3,897,258	14	525,000
10		Taqtaq	479,864	3,947,315	15	106,700

**Table 2:** The results of the statistical summary for the field survey and DEM data.

No.	Name	RMSE	MAE	*R* ^2^
1	Kolak	252,037	199,117	0.989
2	Bneslawa	294,429	195,119	1.000
3	Klkan	80,874	63,664	0.999
4	Mzwryan	13,590	6,906	0.995
5	Tarin	114,715	88,181	0.993
6	HamzaRomi	136,561	69,496	0.983
7	Sarchnar	102,052	48,582	0.997
8	Nwrey Serw	579,977	263,529	0.995
9	Zarda	1,119,112	788,841	0.992
10	Taqtaq	252,037	199,117	0.989

**Fig. 2:**
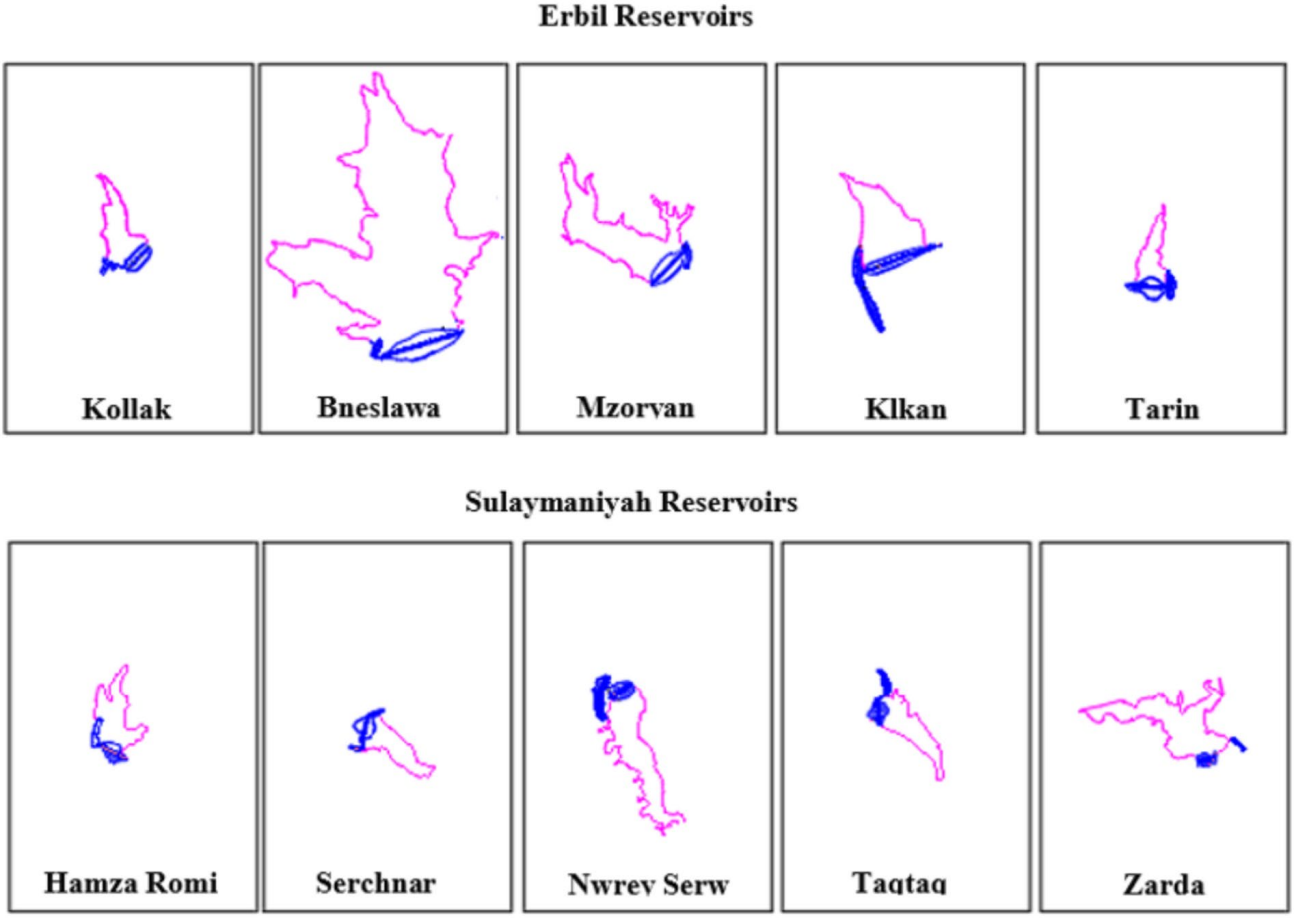
Erbil and Sulaymaniyah reservoirs, top view, with small dam and spillway structures using the same map scale (from survey data).

The reservoirs vary in volume, surface area, height, length, shape, and topography (Fig. 2)^20^. Furthermore, the heights of the small dams vary depending on factors such as designer preferences, site suitability, and local water demand. The maximum capacity of small-dam reservoirs shows notable disparities across geographic locations. Taqtaq Dam in Sulaymaniyah registers a reservoir volume of 106,700 m^3^, whereas the Bneslawa Dam in Erbil boasts a maximum volume of 2,200,000 m^3^ (Table 2).

Concurrently, the height of the small dams ranges from 10 m to 20 m. Notably, the lower-lying small dams are in Erbil, whereas those in Sulaymaniyah tend to be slightly higher. This discrepancy in height is attributed to the general topographical differences between the two regions, with Erbil featuring flatter landforms than Sulaymaniyah’s more undulating terrain^21^.

## Materials and methodology

**Fig. 3:**
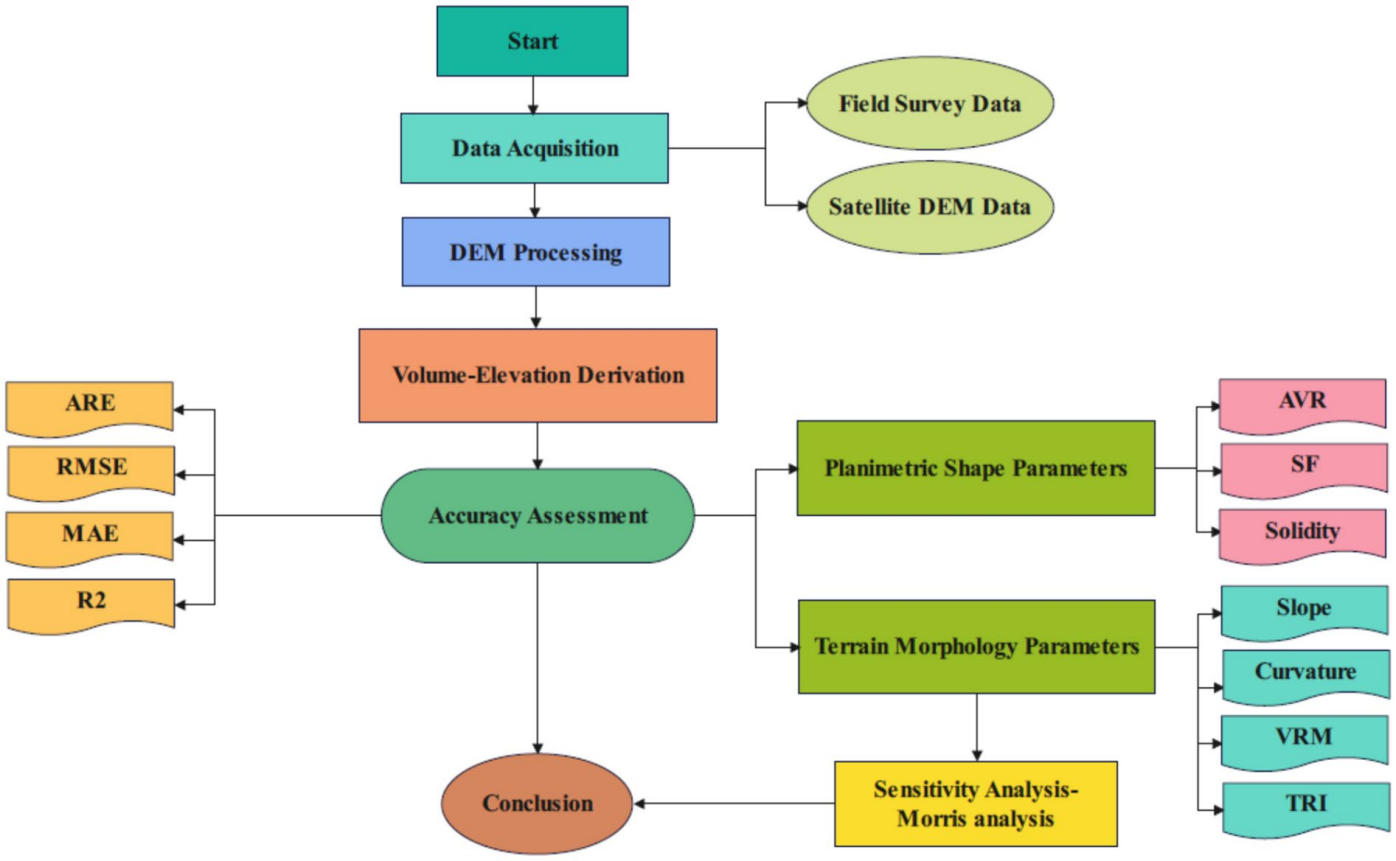
Methodology flowchart.

This study employed a comparative methodology to evaluate the accuracy of one-arc-second Digital Elevation Models (DEMs) against high-precision field survey data for estimating reservoir volume-elevation relationships at small dam sites. The methodological framework consisted of four main phases: (1) Data Acquisition, (2) Data Preprocessing and Volume Calculation, (3) Terrain Metric Extraction, and (4) Accuracy Assessment and Sensitivity Analysis. The methodology flowchart is given in Fig. 3.

### Data acquisition

Data were acquired for this study from two primary sources:

- **Field Survey Data**: High-accuracy topographic data were obtained from the General Irrigation Directorate for all ten reservoirs. The data collection was performed using a Leica GS18 T model, which has a centimetre-level horizontal accuracy of 8 mm + 1 ppm and vertical accuracy of 15 mm + 1 ppm for the RTK technique. These surveys utilised single-baseline RTK with a nearby GNSS base station, set up to use single-baseline RTK with a nearby GNSS base station positioned on predefined, corrected control points from the same directorate.
- **Satellite DEM Data**: One-arc-second (~ 30 m resolution) DEMs were downloaded from the Shuttle Radar Topography Mission (SRTM) database via the USGS Earth Explorer platform for each reservoir location.

### Data preprocessing and volume calculation

The volume–elevation (stage–storage) relationship for the dams was established in ArcGIS using the Spatial Analyst Supplemental Tools. The procedure was carried out as follows:

- **Preprocessing of Elevation Data**: A one-arc-second resolution DEM covering the dam and reservoir areas was first prepared. The dataset was projected into the appropriate coordinate system, clipped to the reservoir boundary, and corrected for artificial depressions by applying a sink-filling operation. These steps ensured topographic consistency and accuracy in subsequent calculations.
- **Application of the Storage Capacity Function**: The Storage Capacity function, also referred to as the Storage–Elevation tool, was then applied to the processed DEM. This tool computes, for each incremental elevation level, the inundated surface area and the cumulative volume stored between the reservoir’s base elevation and the specified stage. In this way, both surface area–elevation and volume–elevation relationships are derived directly from the DEM.
- **Derivation of the Volume-Elevation Curve**: The mentioned tool outputs elevation, surface area, and cumulative volume values. These outputs were subsequently exported to Microsoft Excel, where the volume–elevation curve was constructed. The resulting curve provides a continuous representation of the reservoir’s storage capacity as a function of water level, which is essential for reservoir operation and hydrologic analysis.

### Terrain metric extraction

To evaluate the accuracy of the DEM-derived reservoir volumes and investigate the causes of estimation errors, a suite of morphometric parameters quantifying planimetric shape and terrain morphology was analysed. The following parameters were calculated for each reservoir:

#### Planimetric shape parameters (reservoir area)

- **Area-to-Volume Ratio (AVR)**: The ratio of the reservoir’s surface area to its volume at a specific elevation. Higher AVR values indicate valley shapes approximating an inverted triangle, which may affect DEM accuracy.
- **Shape Factor (SF)**: A dimensionless parameter integrating length, width, area, and perimeter to describe form complexity. An SF of 1.0 indicates a perfect circle, 0.785 a square, and values > 1.0 denote elongated shapes.
- **Solidity**: The ratio of the polygon area to the area of its convex hull, quantifying boundary irregularity. Values approaching 1.0 indicate smoother, more convex shapes. These parameters were calculated using Open JUMP GIS software and the imported tools PolyMorph-2D for Morphometric Analysis, which is used to calculate the Shape Factor (SF) and Solidity^22^, and Maximum Inscribed Circle (MICGIS)^23^.

#### Terrain morphology parameters (5 km buffer Zone)

The spatial variability of the surrounding terrain was characterised using the standard deviation of key topographic metrics derived from the DEM within a 5 km radius of each reservoir centre. First, the DEM was clipped to 5 km circles at the original DEM resolution (one-arc-second), reprojected to the UTM coordinate system, and processed in ArcMap to calculate terrain metrics. Since these metrics are derived from the One-arc-second (~ 30 m resolution), they also inherit the same resolution.

- **Slope**: Variability in the steepness of the terrain surface. Slope is a measure of a surface’s degree of inclination, calculated from the change in elevation between a cell and its neighbouring cells within a digital elevation model^24^.
- **Curvature**: Variability in the concavity or convexity of the landform. Curvature is a measure of the rate of change of slope across a surface, indicating the concavity or convexity of the terrain^24^.
- **Vector Ruggedness Measure (VRM)**: Variability in terrain ruggedness, based on the dispersion of surface normal vectors. It is a vector-based measure that characterises terrain ruggedness by analysing the orientation or alignment of terrain vectors within a defined area^25^. VRM is calculated by decomposing the surface gradient into its horizontal and vertical components and then measuring the dispersion of the vectors’ orientations. VRM was determined using Geomorphology VRM from the Benthic Terrain Modeller (BTM) Tools, which are imported into the ArcGIS toolbox.
- **Terrain Ruggedness Index (TRI)**: Variability in local elevation differences, indicating surface roughness. TRI is a scalar measure that represents terrain ruggedness based on the changeability of elevation values within the study area defined by the masked DEM^26^. TRI was determined using ArcHydro Tools in Python^27^, imported into the ArcGIS toolbox provided by the Esri Water Resources Team.

### Accuracy assessment and sensitivity analysis

#### Accuracy assessment

A comprehensive statistical analysis was conducted to quantify DEM accuracy and rigorously identify systematic biases. The following error metrics were calculated between DEM-derived and field-surveyed volumes at common elevations:

**Table:** 

**Statistical metrics** 1. Absolute Relative Error (ARE) ARE = (1/n) Σ |(O_i_ − P_i_)/O_i_| ……(1) 2. Root Mean Square Error (RMSE) RMSE = √[(1/n) Σ (O_i_ − P_i_)²] ……(2) 3. Mean Absolute Error (MAE) MAE = (1/n) Σ |O_i_ − P_i_| ……(3) 4. Coefficient of Determination (R²) R² = 1 − [Σ (O_i_ − P_i_)²]/[Σ (O_i_ − Ō)²] ……(4)	**Where** ● O_i_: Observed value at data point i (from field survey) ● P_i_: Predicted value at data point i (from DEM) ● Ō: Mean of observed values ● n: Total number of data points ● Σ: Summation over all data points

#### Sensitivity analysis

A sensitivity analysis (SA) was performed to determine which terrain parameter most significantly influences the absolute relative error (%) in volume estimation. The sensitivity analysis was performed using the Morris method:

- **Decision Tree Meta-Model**: Morris sensitivity analysis requires a functional input-output relationship. Since no explicit equation exists for our data, a Decision Tree surrogate model was trained to approximate this mapping, enabling the assessment of each parameter’s influence. The Decision Tree Regression approach was implemented using Python for predictive modelling^28^. For the model optimisation, both Grid Search and Cross-Validation were used from Scikit-learn.
- **Morris Method Implementation**: The sensitivity analysis was conducted using the Morris method^29,30^, which is a global SA approach, implemented through the SALib library in Python. The absolute relative error (%) was treated as the model output. The input factors were the standard deviations of the terrain parameters (Elevation, Slope, Curvature, VRM, TRI) derived from the 5 km territory analysis.
- **Analysis Execution**: After training the model, the sensitivity analysis was performed multiple times to obtain stable results. The sensitivity index values (µ*) from the Morris Method were interpreted to rank the parameters by their influence on the volume estimation error.

## Results and discussions

This study primarily focuses on comparing field data and satellite data. The outcome of the first processing step produced line charts showing the reservoir volume curves for both field surveying and the DEM. Further processing of the DEMs was followed to provide explanations of the landform shape and complexity using various measures. Two main processes were carried out to compare the dam terrain morphology and the differences between the two territories in which the dams were located by taking a 5 km range from the approximate center of each dam. Further processing and analysis were performed for the dam’s 5 km range territory.

### Volume elevation data

**Fig. 4:**
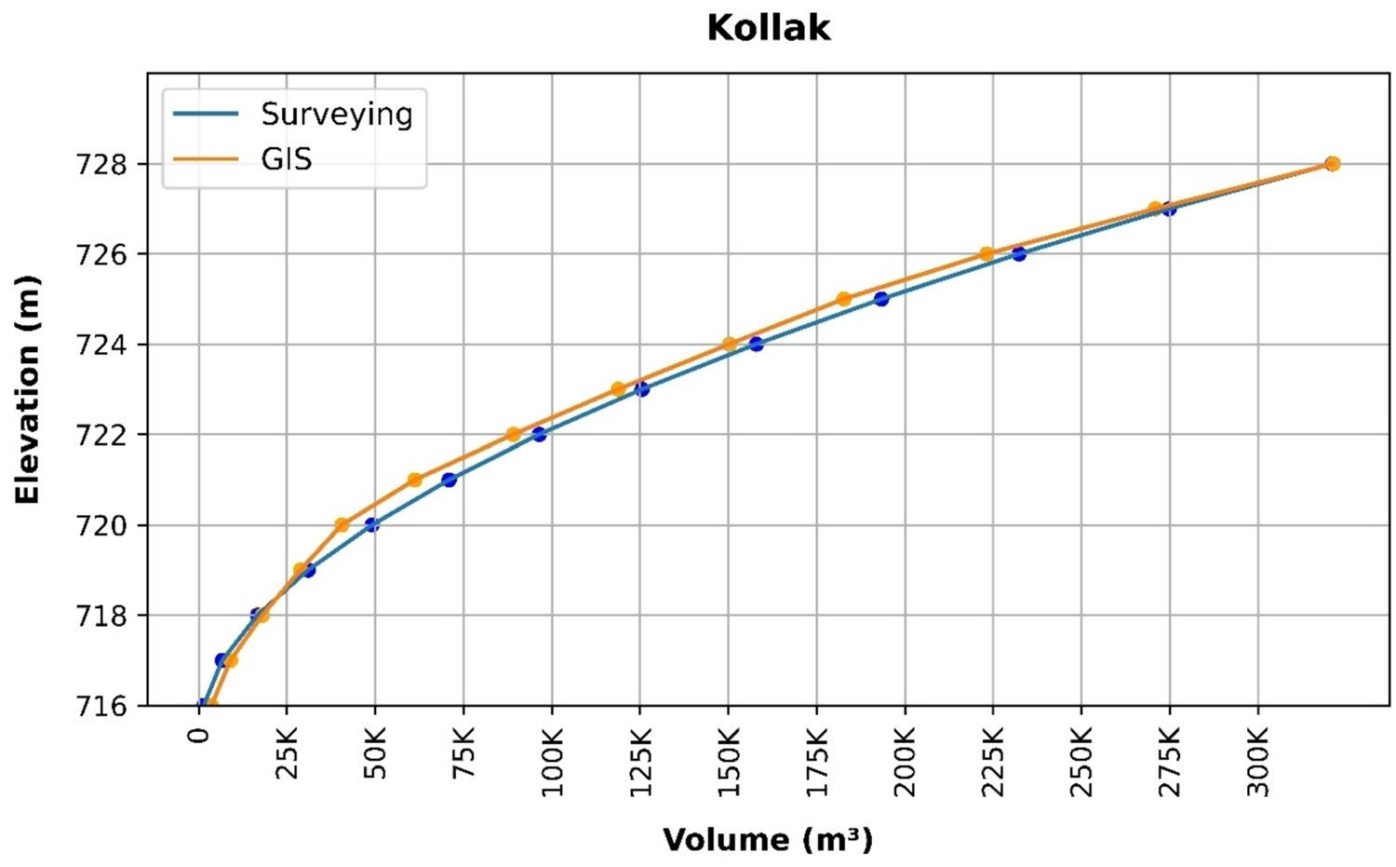
Kollak Volume-Elevation data from the Survey and GIS application.

**Fig. 5:**
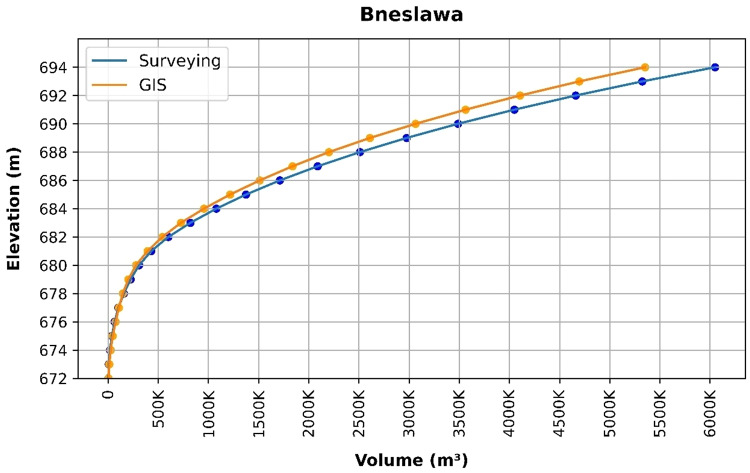
Bneslawa Volume-Elevation data from the Survey and GIS application.

**Fig. 6:**
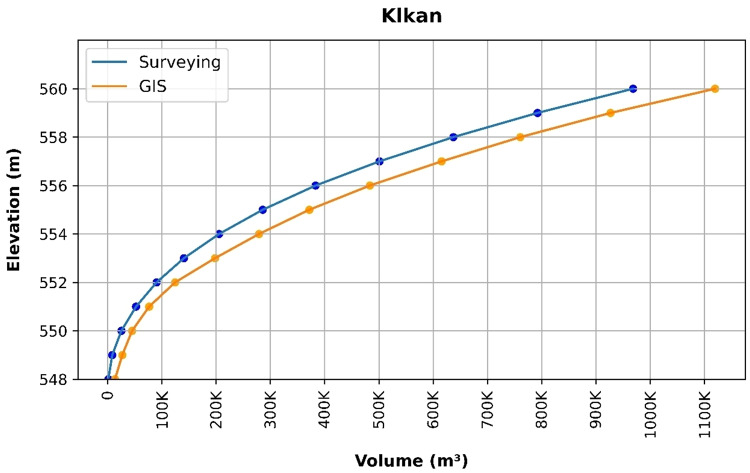
Klkan Volume-Elevation data from the Survey and GIS application.

**Fig. 7:**
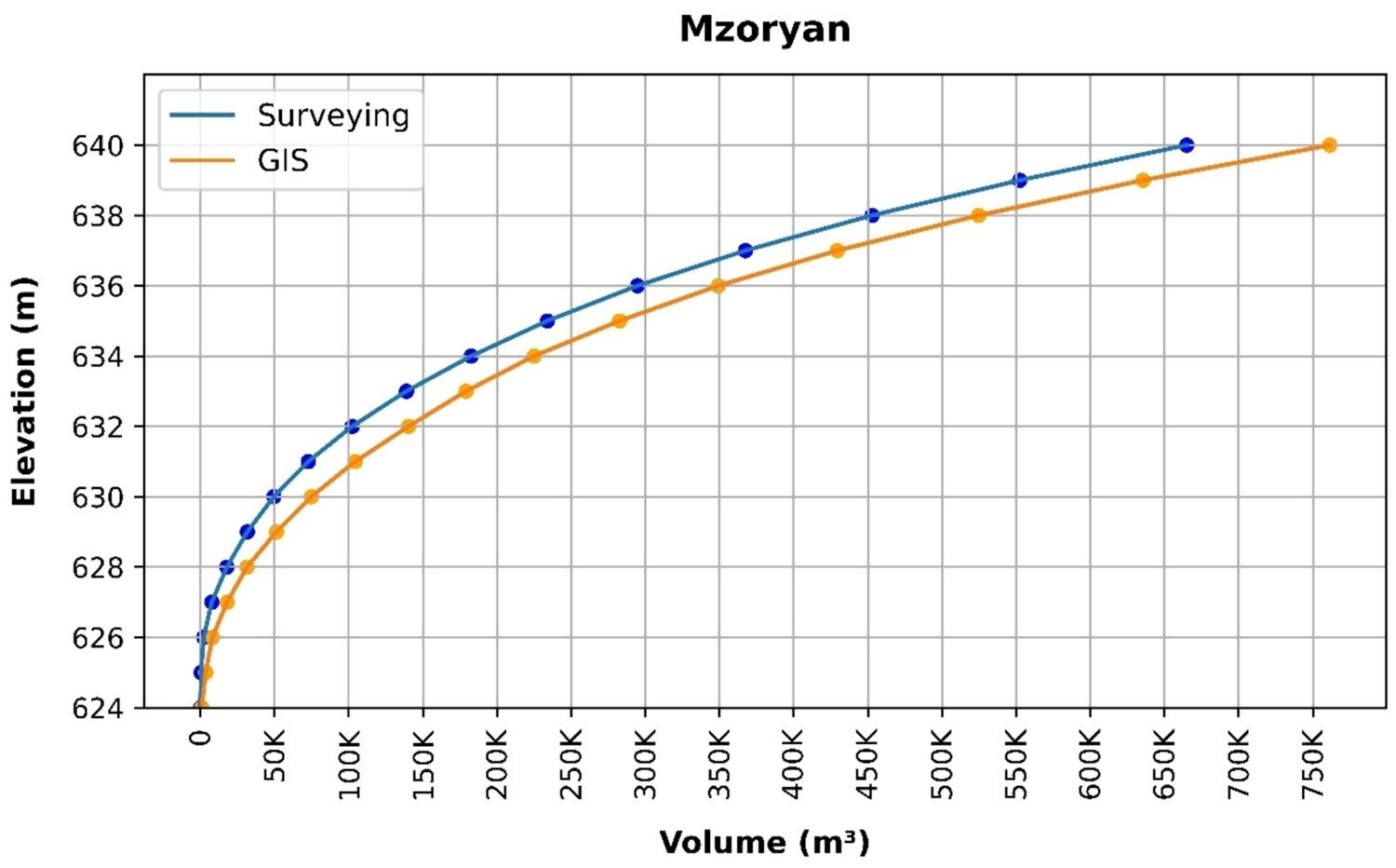
Mzoryan Volume-Elevation data from the Survey and GIS application.

**Fig. 8:**
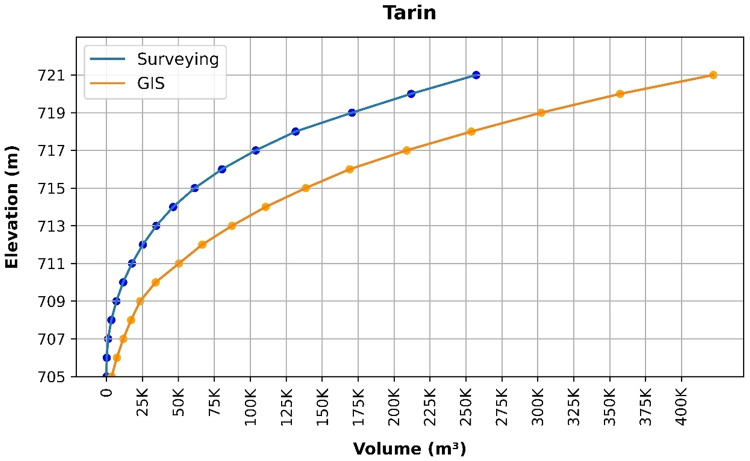
Tarin Volume-Elevation data from the Survey and GIS application.

**Fig. 9:**
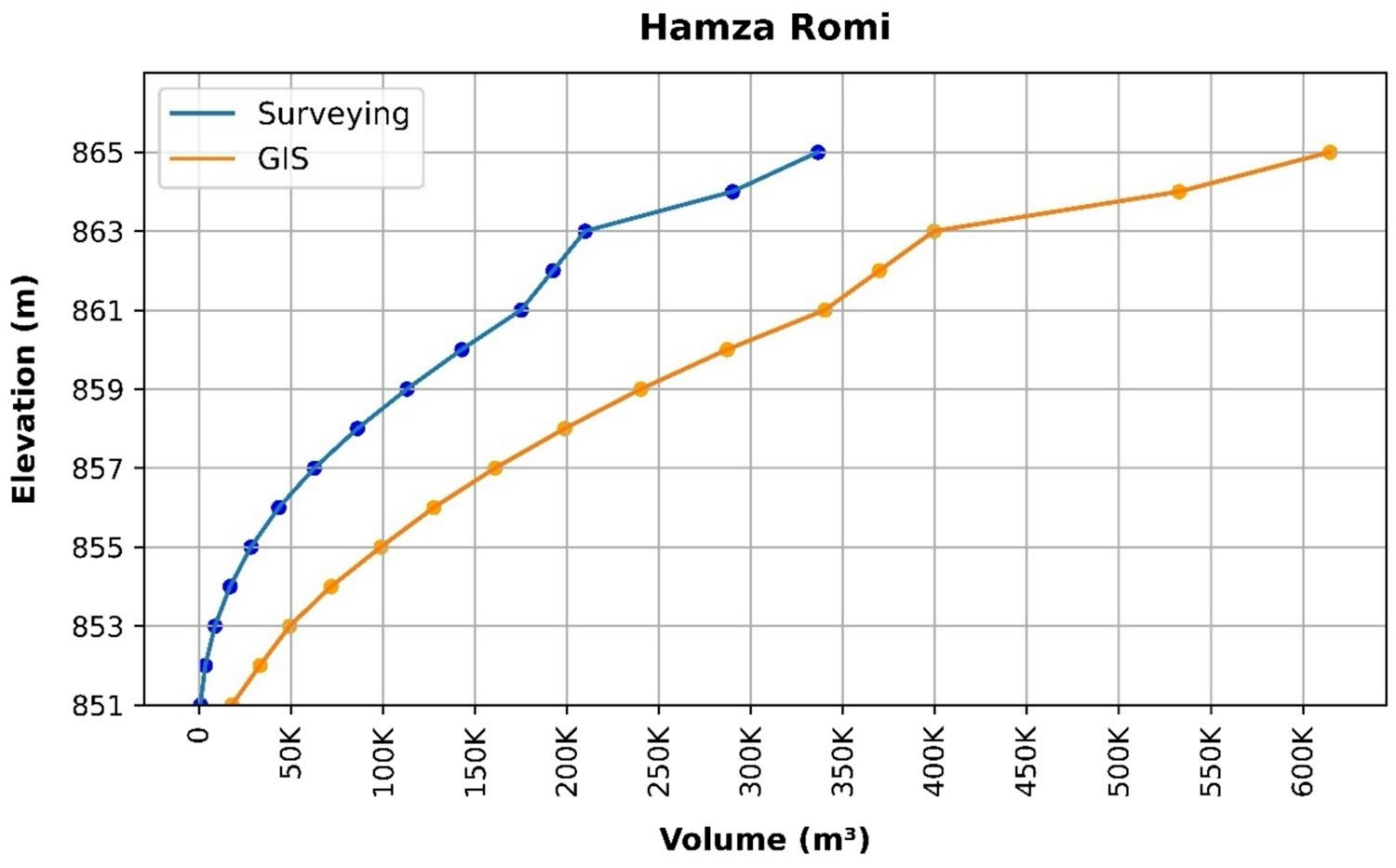
Hamza Romi Volume-Elevation data from the Survey and GIS application.

**Fig. 10:**
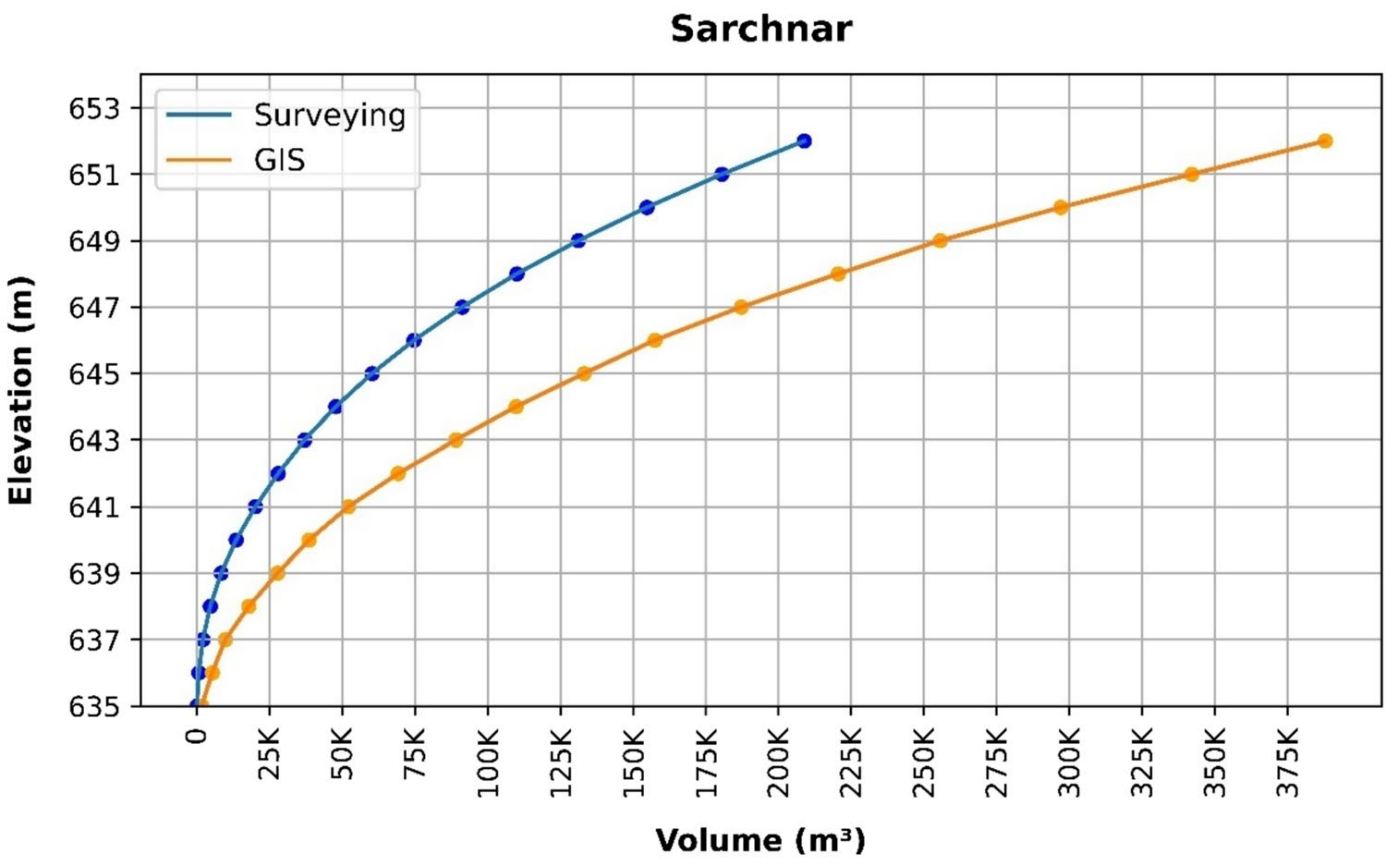
Sarchnar Volume-Elevation data from the Survey and GIS application.

**Fig. 11:**
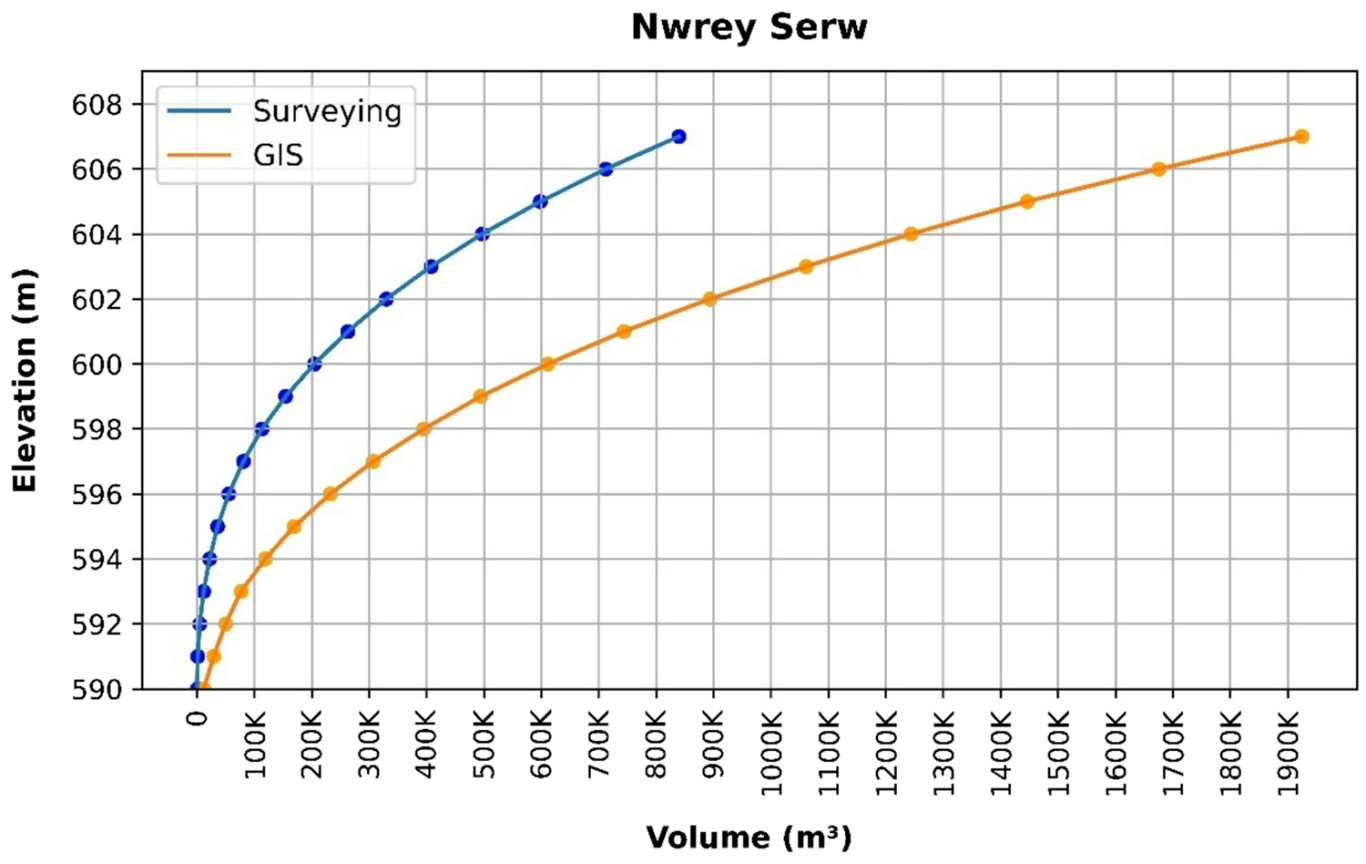
Nwrey Serw Volume-Elevation data from the Survey and GIS application.

**Fig. 12:**
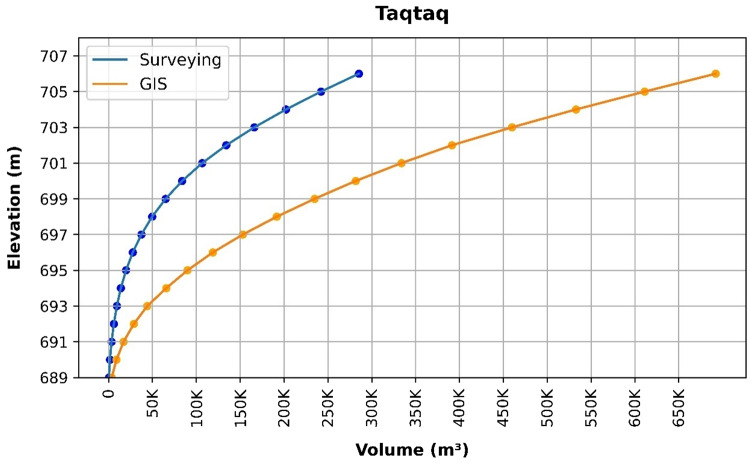
Taqtaq Volume-Elevation data from the Survey and GIS application.

**Fig. 13:**
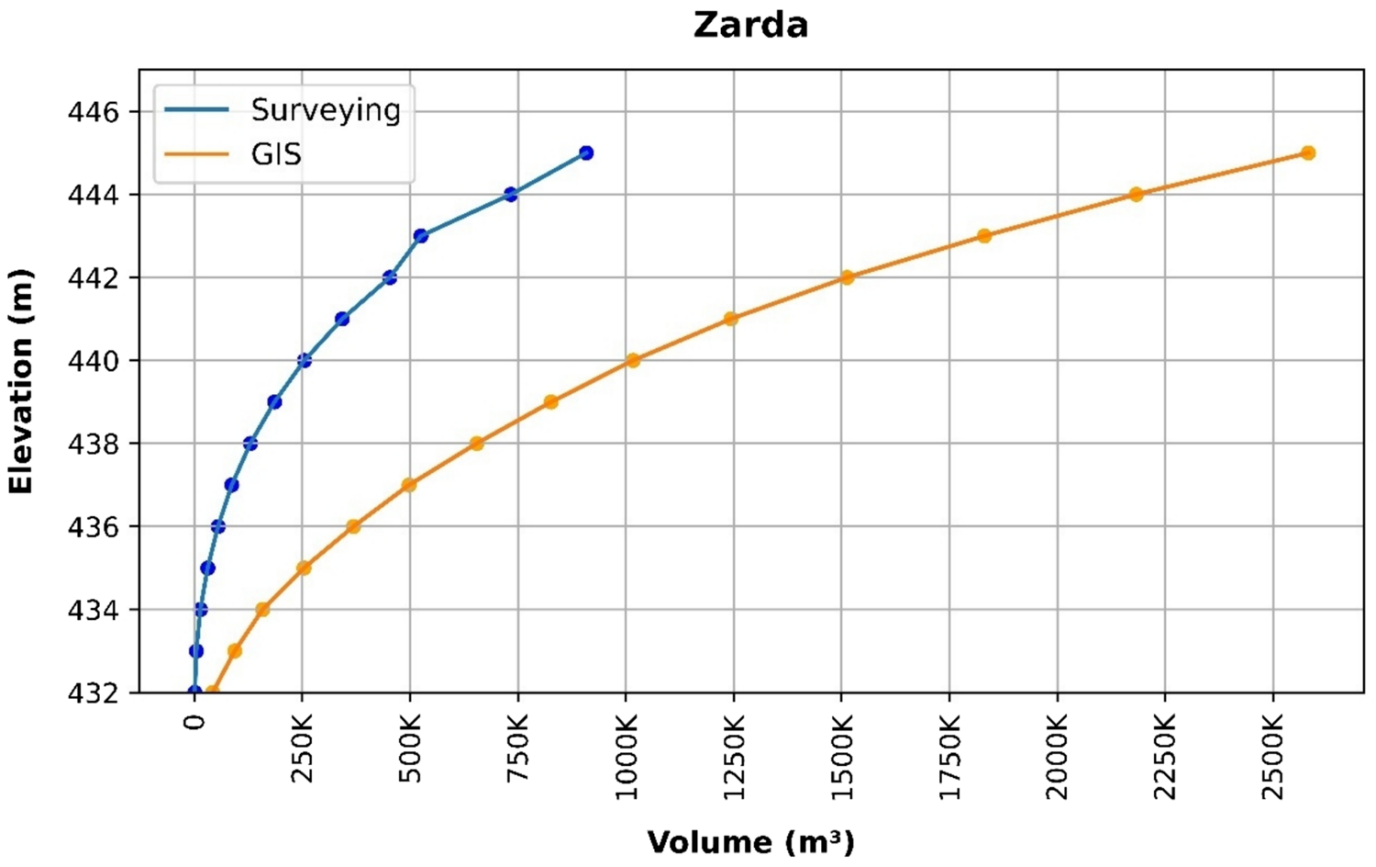
Zarda Volume-Elevation data from the Survey and GIS application.

**Fig. 14:**
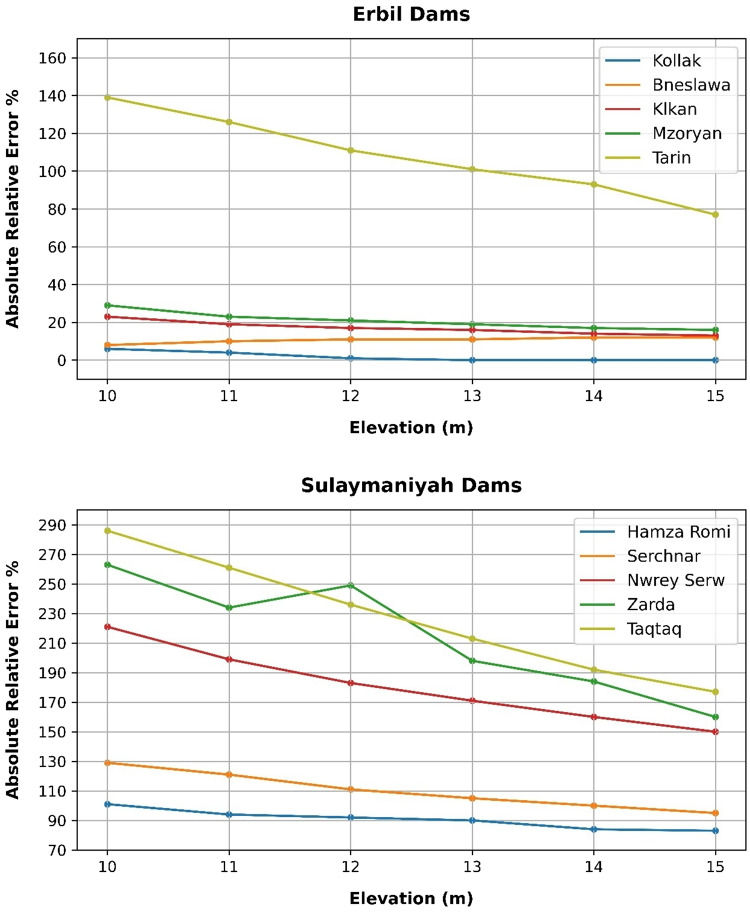
Erbil and Sulaymaniyah dams’ absolute and relative error percentages.

The storage volume of each reservoir was determined using survey data collected at **10 elevation points** and analysed through a GIS application. For each reservoir, these elevation–volume points were used to establish the storage-elevation relationship, and the resulting curves are presented in Figs. 4, 5, 6, 7, 8, 9, 10, 11, 12 and 13. To evaluate the accuracy of the computed volumes, a statistical comparison was performed using all 10 points for each reservoir, and the distribution of errors is summarised in Fig. 14, which shows both absolute and relative error percentages for the Erbil and Sulaymaniyah dams. This analysis demonstrates the reliability of the calculated reservoir volumes, particularly within the dam height range of 10 to 20 m, which is the focus of the study.

The volume-elevation relationships derived from the SRTM DEM exhibit strong structural agreement with survey data (R² > 0.98), confirming the DEM’s ability to accurately capture the general reservoir morphology (Figs. 4, 5, 6, 7, 8, 9, 10, 11, 12 and 13). However, absolute volumetric errors are substantial and highly variable (MAE: 6,906–788,841 m³), revealing a critical limitation for precise capacity estimation.

This discrepancy arises because the ~ 30 m DEM resolution accurately models storage trends but cannot resolve fine-scale bathymetric details essential for precise volumetry. The stark contrast between the low errors in Erbil’s flat terrain and the high errors in Sulaymaniyah’s rugged landscape (Fig. 14) suggests that terrain complexity is the primary driver of errors.

The magnitudes of the RMSE and MAE values are considerable compared with the reservoir storage capacities, which range from approximately 100 to 2,000,000 m³. Given this disparity, these metrics were deemed insufficiently representative for assessing the accuracy of the DEM-derived volumes. Consequently, additional validation parameters were employed to ensure a more reliable evaluation of reservoir storage estimation.

### Dam terrain complexity

To compare the reservoirs, several measures were calculated, accounting for both the planar shape and morphology of the reservoirs (Table 3). The planimetric shape parameters were calculated from the ponds’ bounding polygon shape using various tools. At the same time, the terrain complexity parameters were estimated by processing DEMs to generate slope, curvature, VRM, and TRI. Then, zonal statistics were performed on the generated raster files of the terrain complexity metrics, including those for the DEMs. For further analysis, standard deviations (SD) of the zonal statistics were chosen. The parameters in the table explain solely the 2D shape and morphological traits of the reservoirs that contribute to complexity. Based on the absolute relative error percentage, the results are sorted from the best fit to the poorest fit.

**Table 3:** The reservoirs’ complexity parameters. The Area, AVR, SF, and solidity determine the planar shape of the reservoir’s area, whereas the other parameters determine the morphology.

Location	Reservoir name	Area (m2)	AVR	SF	Solidity	Elevation SD (m)	Slope SD (°)	Curvature SD	VRM SD (m)	TRI SD (m)	Absolute relative error (%)
**Erbil**	Kollak	35,318	0.140	0.778	0.744	3.49	3.88	0.27	0.0014	2.86	0
	Bneslawa	477,186	0.195	0.198	0.605	4.60	4.02	0.28	0.0006	2.78	12
	Klkan	125,575	0.172	0.925	0.832	2.75	3.25	0.25	0.0003	2.17	13
	Mzoryan	109,878	0.182	0.209	0.512	4.37	5.41	0.35	0.0011	3.54	16
	Tarin	30,609	0.161	0.745	0.769	3.53	4.93	0.42	0.002	2.78	77
**Sulaymaniyah**	Hamza Romi	41,372	0.136	0.535	0.667	5.47	6.33	0.50	0.0013	3.84	83
	Serchnar	32,785	0.137	0.906	0.779	5.38	7.91	0.43	0.0014	3.97	95
	Nwrey Serw	73,376	0.147	0.439	0.587	3.79	7.20	0.57	0.005	4.89	150
	Zarda	126,456	0.145	0.225	0.560	3.65	3.35	0.33	0.0006	2.29	177
	Taqtaq	45,275	0.149	0.796	0.691	4.71	4.17	0.51	0.0009	2.57	160

Based on the results, the parameters do not show a positive or a negative trend in the absolute relative error percentage. This is mainly due to the low resolution of the DEM and the small area of the reservoirs. The nearly 30 m resolution of the DEM makes the terrain roughness smoother during processing and averaging elevation values. A study by^31^ investigated the vertical accuracy of the SRTM DEM by analysing three categories: overall, land cover, and slope accuracies, using various statistical methods. Based on that study, we can summarise the vertical accuracy of the used DEM. Table 4 shows that resolution degradation has a more significant effect on steeper terrain and smaller areas. Since dam valleys are small in area, the approach of processing and analysing reservoir areas does not provide insight; thus, the reservoir territory approach was adopted.

**Table 4:** SRTM DEM error propagation.

Metric	Overall	Slope 5–10	Slope 10–15	Slope 15–20	Slope 20 − 15	Slope > 25
Median (m)	1.65	2.1	2.5	3	3	4
NMAD (m)	3.65	4	4.5	5.5	6	7.7
RMSE (m)	5.38	5.5	7	8	8.5	10
LE95 (m)	11.19	12	14	17	18.5	22

### Dam’s territory terrain complexity

Since the reservoir-specific parameters, both planimetric and morphological, failed to explain the discrepancies between the actual and DEM-calculated volumes (Table 3), a territorial analysis was performed. Moreover, the study focuses on small dams with a smaller surface area. Thus, bathymetric analysis of the dams themselves cannot provide sufficient information when using a low-resolution DEM, such as the SRTM DEM.

In addition, the difference in impact between the two approaches (dam and terrain analysis) is justified by comparing the reservoir surface area (planner analysis) and the absolute relative error (%) for the Erbil and Sulaymaniyah regions. The results reveal significant differences, despite nearly equivalent areas (Table 3). For example, Kollak in Erbil, with a surface area of 35,318 m^2^, has 0% error. Meanwhile, Serchnar in Sulaymaniyah, with a surface area of 32,785 m^2,^ has a 95% error. Also, no significant differences or clear patterns were found in the remaining parameters using the dams’ terrain analysis (Table 3). Therefore, this approach of territory terrain complexity analysis was adopted.

This approach relies on the fact that the geomorphology of the reservoir’s surrounding terrain generally reflects the ruggedness of the reservoir itself. From the approximate center of each reservoir, a 5 km range DEM was extracted and processed. The range was selected to cover the largest reservoir boundary without the reservoir 2D polygon being circumscribed by a circle. The circular zone with a 5 km radius was determined based on two significant factors:

1. There are differences in the surface areas of the reservoirs, ranging from a minimum of ~ 0.03 km² to a maximum of ~ 0.48 km². However, the circular buffer zone has a significant surface area of ~ 78.5 km², compared to the small dams; thus, the discrepancies found between the dams are negligible compared to the larger area of the territorial zone.
2. The proposed 5 km buffer radius adequately captures the relevant topographic influences, as local terrain features such as minor valleys and ridgelines within this domain exhibit negligible morphological variation relative to the broader reservoir environment, specifically using a low-resolution DEM. The geomorphological characteristics of the reservoir surrounding territories are thus representative of the dam terrain complexity at this spatial scale.

Like the reservoir terrain complexity results, most parameters do not exhibit a trend in the territorial complexity analysis, except for TRI, which shows a significant positive correlation with the percentage of absolute relative error (Table 5). The results indicate that the first five reservoirs in Erbil have TRI measures below 0.1, whereas those in Sulaymaniyah are above 1.0 on a large scale. The TRI suggests that the first (5 km) territorial DEMs (located in Erbil) have lower roughness; therefore, the low-resolution DEM has a lesser impact on the accuracy of the results in these regions. In contrast, the other reservoirs in Sulaymaniyah are highly affected by the DEM resolution. This can be justified by the statistical analysis found in Table 4. This is why the SRTM DEM provides more accurate results for Erbil dams than for Sulaymaniyah dams.

**Table 5:** The reservoirs’ territory complexity parameters, which range up to 5 km, are centered on the reservoirs.

Location	Reservoir	Elevation SD (m)	Slope SD (°)	Curvature SD	Plan SD	Profile SD	VRM SD	TRI SD (m)	Absolute relative error (%)
Erbil	Kollak	117.454	3.39	0.584	0.308	0.169	0.004	0.001	0
	Bneslawa	52.793	7.57	0.588	0.314	0.369	0.003	0.004	12
	Klkan	51.717	8.82	0.285	0.151	0.369	0.001	0.006	13
	Mzoryan	74.379	6.26	0.487	0.257	0.304	0.002	0.003	16
	Tarin	115.307	8.63	0.556	0.295	0.350	0.003	0.005	77
Sulaymaniyah	Hamza Romi	149.123	13.43	0.572	0.295	0.371	0.003	13.436	83
	Serchnar	146.257	12.03	0.755	0.388	0.491	0.005	12.031	95
	Nwrey Serw	61.937	6.80	0.493	0.256	0.315	0.002	6.8	150
	Zarda	37.822	5.29	0.480	0.256	0.292	0.002	5.298	160
	Taqtaq	57.382	6.89	0.469	0.244	0.300	0.002	6.894	177

### Sensitivity analysis

To further study the impact of territorial parameters on the relative absolute error percentage, a sensitivity analysis^8^ was performed using the Morris method. Input ranges were defined by setting parameter bounds to the dataset’s observed minimum and maximum values. The sampling design utilised the SALib library, employing 450 trajectories and 20 levels per parameter.

A Decision Tree Regressor, optimised via Grid Search with Cross-Validation, was used as the meta-model. The SA process was repeated 50 times with random seeds, and the minimum sample split for the Decision Tree Regressor was found to be 6. The model was also manually evaluated with a smaller minimum sample split of 5 values. For each sample split and parameter (each iteration), an SA value was computed, and the average was then calculated for each parameter. The final sensitivity indices (µ*), presented in Table 6, are the average of these 50 replications.

**Table 6:** The result of the sensitivity analysis for the reservoirs’ territory. The values presented are averages across 10 seeds for two sample splits, 5 and 6.

Samples split	Elevation SD	Slope SD	Curvature SD	Plan SD	Profile SD	VRM SD	TRI SD
5	8.44	24.03	18.26	10.16	17.84	16.30	83.42
6	0	0	0	0	0	0	86.65

**Fig. 15:**
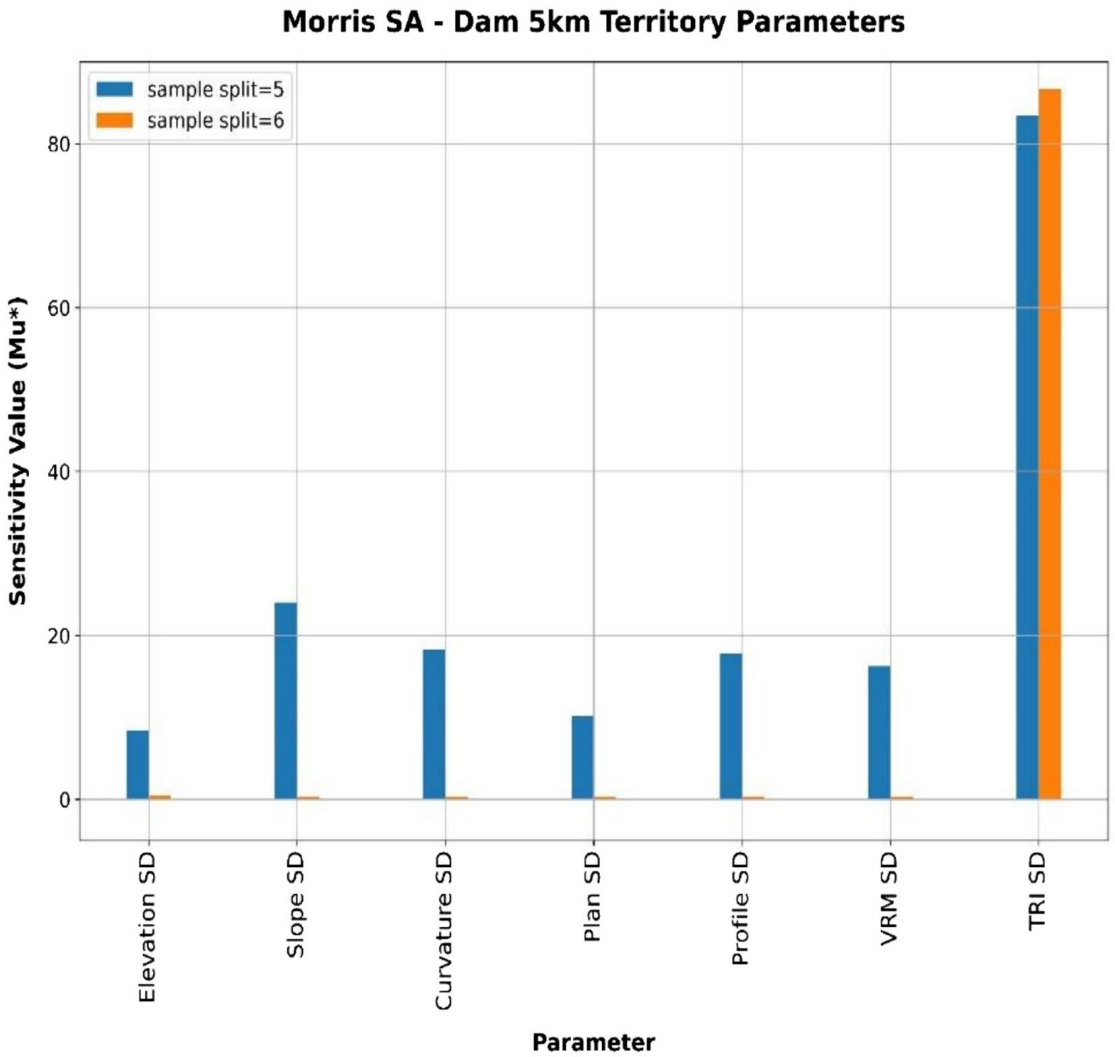
Morris Sensitivity Analysis for the dam’s territories complexity parameters using an average of 50 seeds for two different sample splits, 5 and 6.

The results indicate that the relative absolute error percentage is susceptible to the TRI, with a mean sensitivity value (µ*) exceeding 80. The other parameters demonstrated a lower influence, with µ* values ranging from approximately 0 to 25, as shown in Fig. 15.

The significantly higher influence score of TRI compared to other parameters in reservoir volume sensitivity analysis stems from its direct quantification of vertical elevation variability, which is the primary geomorphological control on reservoir storage capacity.

TRI calculates the root-mean-square elevation deviation within a defined neighbourhood for each cell, effectively capturing the amplitude of relief and valley depth that determine the physical space available for water storage. In contrast, VRM measures variation in slope and aspect orientation, which describe surface texture but decouple ruggedness from absolute elevation differences^32,33^. Additionally, slope indicates a change in the terrain’s steepness, and curvature (plan and profile) measures reveal variability in the landform’s concavity or convexity. These measures are derived from elevation data but are less directly tied to water volume calculations^34^.

**Fig. 16:**
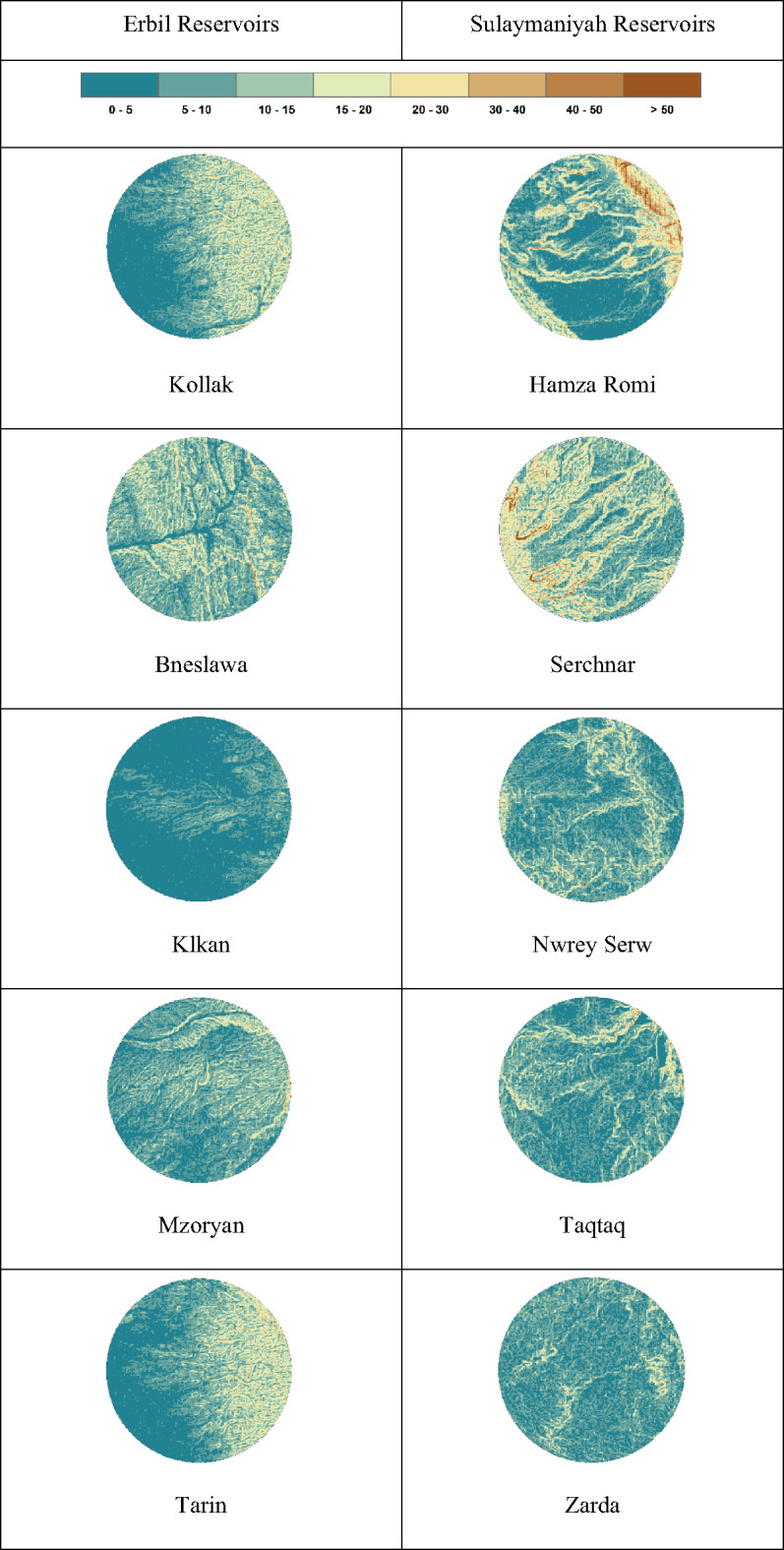
The Mapped TRI of the dams, categorized by the same classification and color scheme.

Based on the chosen 5 km territory complexity parameters and SA results, since TRI has a significant correlation with the absolute relative error (%), TRI can be used to decide whether the calculated reservoir volume is acceptable. From Table 3, if the TRI value is below 0.01, we can consider the computed volume to have an absolute relative error of less than 20%, which is true for four dam locations: Kollak, Bneslawa, Klkan, and Mzoryan. In the case of Tarin, which resulted in an absolute relative error of 77%, this could be related to its surface area, as shown in Fig. 16, which illustrates the differences in the dams’ TRI between Erbil and Sulaymaniyah cities, ordered from best fit to worst fit.

Whereas conventional DEM validation (e.g., Talchabhadel et al.; Meadows et al.) ranks products based on vertical accuracy, this study establishes that terrain ruggedness (TRI), rather than aggregate vertical error, dictates DEM suitability for reservoir volumetry. For small reservoirs, volumetric accuracy is compromised by the systematic smoothing of valley geometry in complex terrain—an integrative error poorly represented by point-based metrics. This shifts the paradigm from a quest for a universally superior DEM to a terrain-dependent suitability framework, providing a predictive tool to define the operational domain of widely available data, such as SRTM, for hydrological planning.

## Conclusions

This study provides the first rigorous, terrain-dependent validation of one-arc-second SRTM DEMs for estimating volume-elevation curves at small dams. The main scientific contribution is a quantitative, predictive framework that directly links volumetric error to the standard deviation of the Terrain Ruggedness Index (TRI) within a 5 km buffer zone, accounting for over 80% of the error variance. This moves beyond simple accuracy reporting to providing a practical decision-making tool.

The findings demonstrate that these freely available global DEMs can be considered as viable alternatives to traditional surveys for preliminary feasibility assessments of small dam projects, particularly in data-scarce regions. Strong correlations between DEM-derived and field-surveyed volumes (R² = 0.983–1.000.983.000) confirm the fundamental structural validity of satellite-based data in capturing reservoir morphology, fulfilling the primary objective of this study. However, a crucial limitation was identified: while DEMs show excellent structural agreement, their volumetric precision exhibits substantial variation (MAE: 6,906 − 788,841 m³; RMSE: 13,590-1,119,112 m³) that correlates strongly with terrain complexity. The Terrain Ruggedness Index (TRI) was found to serve as the primary predictor of DEM accuracy, explaining over 80% of the error variance, according to Morris’s sensitivity analysis, thereby achieving the second objective by providing a clear geomorphological explanation for the observed error patterns. This relationship was clearly manifested in the comparative study between the flat terrain of Erbil (TRI SD < 0.1, errors < 20%) and the rugged topography of Sulaymaniyah (TRI SD 5.298–13.436, errors > 150%).

The key methodological innovation lies in the integrated use of territorial terrain analysis and global sensitivity analysis. By shifting the focus from the reservoir’s own geometry to the geomorphology of its surrounding territory and by employing the Morris Method, we successfully identified TRI as the primary predictor of accuracy—an approach more effective than reservoir-specific planimetric indices.

This approach, combined with the sensitivity analysis framework, provides water resource planners with a practical tool for assessing pre-project accuracy and fulfilling the third objective by demonstrating that the standard deviation of TRI explains over 80% of the error variance. This finding is not merely correlative; it identifies TRI as the key mechanistic link between terrain character and DEM performance. The results indicate that one-arc-second DEMs yield sufficient accuracy for preliminary planning in low-TRI regions, while high-TRI areas require either higher-resolution data or field verification.

Finally, the fourth objective, to establish clear applicability thresholds, was achieved. It is demonstrated that SRTM DEMs provide sufficient accuracy for preliminary planning (errors < 20%) in low-ruggedness terrains (TRI SD < 0.1) but become highly unreliable (errors > 150%) in rugged landscapes (TRI SD 5.298–13.436). This TRI-based threshold provides the intended predictive framework, enabling the suitability of SRTM data to be pre-assessed for proposed dam sites using readily derived terrain metrics.

The methodologies presented here could be significantly enhanced by integrating modern geospatial technologies. It is recommended that future work explore the potential of AI-based DEM correction. Furthermore, the fusion of satellite DEMs with high-resolution LiDAR and photogrammetry data would provide a much finer representation of critical terrain features. Adopting these techniques would bridge the gap between large-scale DEM analysis and site-specific accuracy.

## Supplementary Information

Below is the link to the electronic supplementary material.

Supplementary Material 1

## Data Availability

All data supporting the findings of this study are available from the author upon request from the corresponding author (nadhir.alansari@ltu.se).
