# Supplementary material for: Assessing SRTM one Arc second DEM accuracy for small dam volume-elevation curves using terrain metrics
Source: Sci Rep. 2025 Dec 27;15:44666. doi: 10.1038/s41598-025-30483-7 (PMC12749584; doi:10.1038/s41598-025-30483-7)
Supplement: Supplementary file 1 — Supplementary Material 1 [file 41598_2025_30483_MOESM1_ESM.pdf]

Dear Editor

The authors of our paper are as follows:

1. Peshawa B. S. Ahmed
2. Nawbahar F. Mustafa
3. Shvan F. Aziz
4. Nadhir Al-Ansari

Best regards.

Peshawa B. S. Ahmed

Nawbahar F. Mustafa

Shvan F. Aziz

Nadhir Al-Ansari
